# Laying Down the Groundwork for an International Measurement Infrastructure

**DOI:** 10.1093/geroni/igab046.554

**Published:** 2021-12-17

**Authors:** Katherine McGilton, Franziska Zuniga, Michael Lepore, Kirsten Corazzini, Charlene Chu

**Affiliations:** 1 KITE-Toronto Rehabilitation, University Health Network, Toronto, Ontario, Canada; 2 Basel University, Ostermundingen, Basel-Stadt, Switzerland; 3 LiveWell Alliance, Southington, Connecticut, United States; 4 University of Maryland School of Nursing, Baltimore, Maryland, United States; 5 University of Toronto, Toronto, Ontario, Canada

## Abstract

The COVID-19 epidemic has brought to light the significant problems in the long-term care (LTC) sector, specifically the lack of an infrastructure to collect and aggregate data between LTC sectors in different countries. This talk will briefly describe goals of the WE-THRIVE initiative, and focus on exploring the development of “workforce and staffing” common data elements for LTC. We will describe how the subgroup is “laying down the groundwork” within this domain with various methodologies to develop CDEs related to workforce and staffing. The CDEs aim to measure staff retention and turnover, evaluating nursing supervisor effectiveness, and staff training in LTC. Anticipated challenges of this international work will also be highlighted. International research on LTC can valuably inform LTC policy and practice, and the proposed CDEs can facilitate data sharing and aggregation internationally, including low-, middle-, and high-income countries.

